# Comparison of classic statistical methods and machine learning approaches to classify readiness

**DOI:** 10.1093/bioinformatics/btag585

**Published:** 2026-08-25

**Authors:** Ashley E Copenhaver, Chris Puglisi, Mohammed A Eslami

**Affiliations:** Netrias, LLC, Annapolis, MD 21401, United States; Netrias, LLC, Annapolis, MD 21401, United States; Netrias, LLC, Annapolis, MD 21401, United States

## Abstract

**Motivation:**

Predicting physical and cognitive readiness in warfighters is critical for mission success. These predictions can be improved by identifying key biomarkers using multiple omics modalities. The MASTR-E study conducted by McKetney and colleagues is one of the most comprehensive multi-omics studies of saliva samples collected from warfighters, which also applied classic linear statistical (CLS) techniques to discover key biomarkers of readiness. Aligning with McKetney et al.’s assumptions, we operationalize readiness as a binary proxy, where pre-mission samples are labeled as “ready” to reflect a rested, unstressed physiological baseline, while post-mission samples are labeled “not ready” to reflect cumulative physical and cognitive load from the mission. As such, readiness here is not a direct biological or physiological construct, but an inferred state likely dominated by stress-related physiological changes. This assumption and definition is discussed further in the Introduction and Limitations sections. Here, we apply machine learning (ML) analyses to better assess generalizability, consider hidden interactions, and identify nonlinear patterns in the data. We investigated whether ML approaches could predict readiness and identify relevant biomarkers. ML models were trained on proteomics-only or metabolomics-only datasets to classify participants as ready or not ready and important model features were considered as putative biomarkers. Training and testing datasets were curated for two objectives: (i) recognize biomolecular signatures indicative of readiness within the same donor and (ii) assess generalizability across warfighters by withholding donors for testing.

**Results:**

Proteomics-based models achieved AUCs of 0.907 ± 0.034 and 0.860 ± 0.063 for Objectives 1 and 2, respectively. Metabolomics-based models achieved Objective 1 AUC of 0.994 ± 0.007 and Objective 2 AUC of 0.993 ± 0.010. Comparative analysis with existing literature validates the model’s feature importances, but the identified putative biomarkers significantly differ from those discovered through CLS analyses, as only one ML-identified biomarker overlapping with those identified through CLS methods. We show that these ML models and identified features are more robust to noise and generalizable across participants than those identified using CLS methods.

**Availability:**

The analysis pipelines are provided as Jupyter notebooks, including all code and documentation, and are available publicly on GitHub at {https://github.com/netrias/ReadinessClassification}.

## 1 Introduction

Factors such as stress can significantly impact an individual’s readiness to perform high-intensity missions). To ensure that warfighters are appropriately selected or omitted from missions accordingly, individuals undergo time-consuming subjective evaluations and limited physiological testing to determine their state of readiness (Caldwell and Caldwell 2005, Lieberman *et al.* 2005). These assessments often rely on biomarkers indicative of high stress levels, such as cortisol, as proxies for readiness (Beckner *et al.* 2022) since the concept of readiness has no universally accepted biological definition. This lack of a clear biological framework makes it difficult to objectively determine whether an individual is mission ready. Furthermore, as readiness is a complex phenotype likely influenced by multiple physiological pathways, the reliance upon solely stress biomarkers provides an incomplete picture for the assessment of individuals. As such, a more comprehensive approach that identifies robust and reliable biomarkers reflective of readiness itself is necessary to improve mission decision-making and overall warfighter performance. Recent technological advances have enabled rapid, non-invasive sampling methods via saliva to capture an individual’s physiological state in real time and undergo integration with algorithmic frameworks to predict disease (Nie *et al.* 2014, SoRelle *et al.* 2020, Melo Costa *et al.* 2021). If reliable biomarkers of readiness can be identified in saliva, this could provide a practical, scalable, and objective tool for assessing warfighter readiness in the field.

Classic linear statistical (CLS) methods such as principal component analyses (PCA) and linear discriminant analysis (LDA) are widely used for biomarker identification; however, CLS primarily detects differences in individual molecular features and may struggle to capture complex, nonlinear interactions between biomarkers. In contrast, machine learning (ML) excels at extracting subtle, high-dimensional patterns that may not be evident through comparative analysis alone but tends to struggle with solution overfitting (Ng *et al.* 2023). Because readiness is a complex physiological state influenced by multiple factors, we hypothesized that CLS and ML approaches may identify reveal different biomarkers. Therefore, in this study, we compared the performance of a CLS approach vs. an ML approach to investigate whether the ML approach could produce models with high readiness predictive accuracy and identify robust biomarkers indicative of readiness.

The dataset used in this study is one of the most comprehensive multi-omics datasets of salivary samples collected from warfighters, containing proteomics and metabolomics data from 20 individuals across up to three phases of a mission: pre-mission (3 days), mission (3 days), and post-mission (4 days) (McKetney *et al.* 2022). The longitudinal design of this study combined with the military mission context, number of donors, and both proteomics and metabolomics makes this dataset uniquely suited for identifying biomarkers, as opposed to other studies focusing on athletes or single modes of data (Zauber *et al.* 2012, Luti *et al.* 2023, Lindsey *et al.* 2025). When this dataset was collected, CLS techniques were applied to successfully identify biomarkers associated with readiness. Linear Discriminant Analysis (LDA) was used to select the top 5 proteins that individually distinguished readiness, and the combined use of these proteins resulted in a model with an area under the curve (AUC) of 0.771.

**Figure 1:**
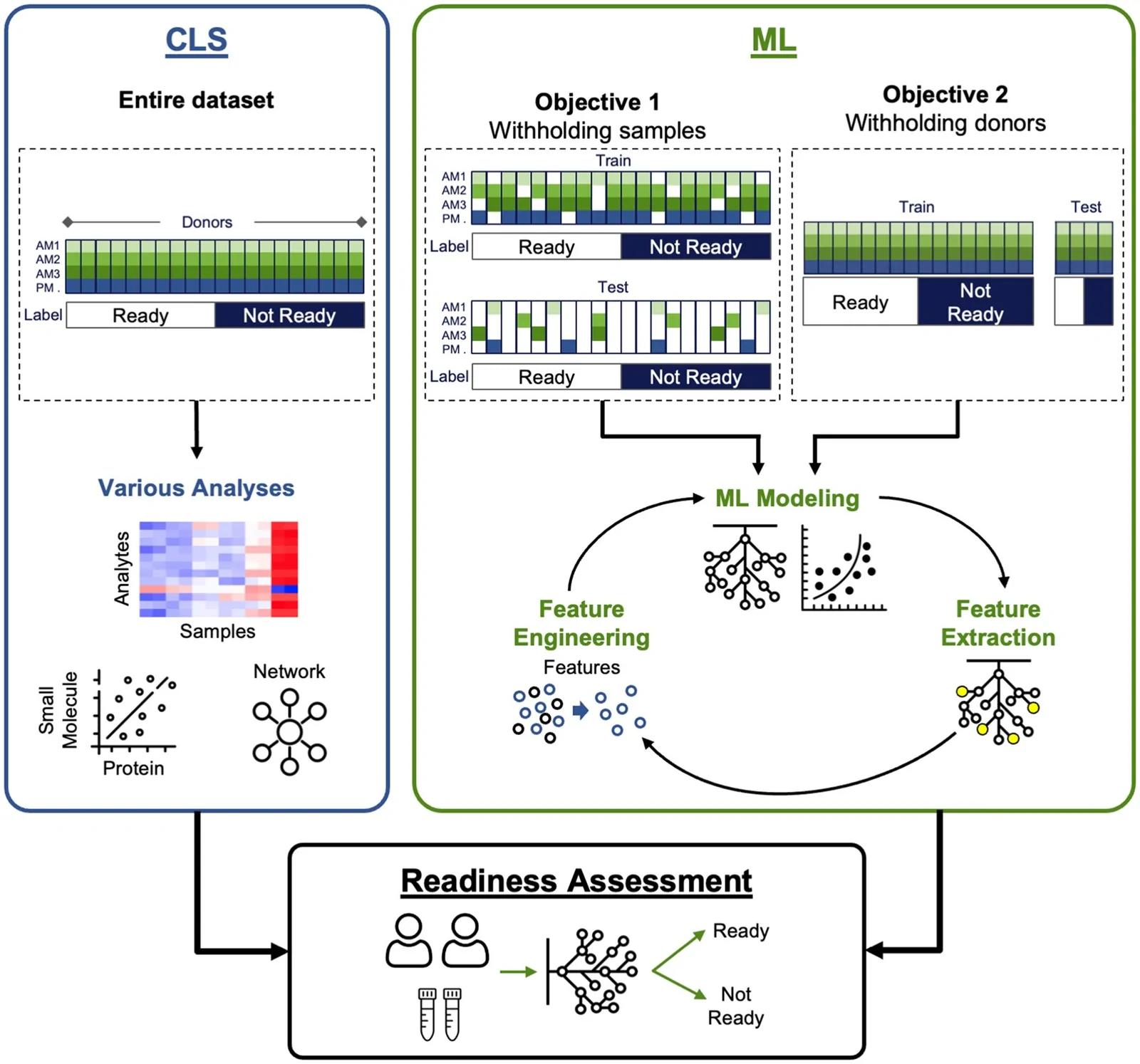
Comparison of CLS analysis pipeline and ML analysis pipeline to identify biomarkers indicative of readiness. AM1, AM2, AM3, and PM refer to timepoint identifiers within each donor.

We present an ML approach to predict readiness based on salivary proteomics and metabolomics data. Although both proteomics and metabolomics data were available, here we present only models that were trained independently on each data type with no integrative multi-omics models. A comparison between our ML approach and CLS analysis is shown in Fig. 1. We defined pre-mission samples as “ready” and post-mission samples as “not ready,” which aligns with the definition of readiness used by McKetney *et al.* (2022). Notably, these readiness labels are binary proxies of an inferred state likely dominated by stress-related physiological changes. Models were trained on either proteomics or metabolomics data to classify an individual as ready or not ready. Training and testing datasets were curated for two objectives: (i) recognize biomolecular signatures indicative of readiness across the same donor, which aligns with the previously published LDA approach (McKetney *et al.* 2022), and (ii) assess generalizability across warfighters. Our results indicate that proteomics ML models achieve AUC of 0.907 ± 0.034 and 0.860 ± 0.063, for Objectives 1 and 2, respectively, while metabolomics ML models achieve AUC 0.994 ± 0.007 and 0.993 ± 0.010, for Objectives 1 and 2, respectively. In comparison to LDA, Objective 1 displays a 13% enhancement in predictive accuracy. Further, we identify novel biomarkers indicative of readiness that were validated through a literature review, but that significantly differed from those found through solely CLS techniques. This work provides a foundation for facilitating real-time readiness assessments in operational settings, providing an objective and data-driven tool for decision-making in the field.

## 2 Methods

### 2.1 Dataset

The data used in this study was previously published by McKetney *et al.* in which authors collected saliva samples from 20 individuals at various timepoints throughout a 10-day event (McKetney *et al.* 2022). There were three phases of the mission: pre-mission, mission, and post-mission.

The mass spectrometry proteomics data was retrieved from ProteomeXchange Consortium (http://proteomecentral.proteomexchange.org) via the PRIDE database with the dataset identifier PXD026680. For this study, all pre-mission and post-mission samples (8 timepoints) from all participants (20 donors) were used.

Metabolomics data was originally presented in McKetney *et al.* (2022) and was retrieved from a hard drive shared by an author of that study. The metabolomics dataset available contained only MS1 scans. For this study, only pre-mission (12 timepoints) and post-mission (14 timepoints) metabolomics data was used. All timepoints had data for at least 50% of donors. Data from 10 timepoints, scattered across donors, was missing.

### 2.2 Preprocessing and bioinformatics

#### 2.2.1 Poorly correlated replicate removal

We removed poorly correlated replicates from the dataset prior to model training. This method aimed to reduce the risk of the ML models learning uninformative signals from noisy samples. To remove said replicates, we reduced the data to two dimensions using principal component analysis, capturing 66% of the variance. For each donor, the eight samples were plotted in two dimensions, and the silhouette score [silhouette_score() from scikit-learn version 1.6.0] was calculated using the pre and post categories as clusters. We quantitatively identified the poorly correlated replicates by capturing those that, when removed, improved the silhouette score of the clusters by 50% or more. This conservative threshold was chosen to capture removals likely to reflect substantial improvements in within-donor cluster consistency, minimizing unnecessary data loss. We identified and removed the 29 poorly correlated replicates from the proteomics dataset. Zero poorly correlated replicates were identified in the metabolomics dataset.

#### 2.2.2 Differential abundance

We analyzed 2064 proteins that passed quality control for differential abundance (DA). Quality control removal included: contaminant identifications (n = 101), reversed-sequence decoys (n = 46), modification-site-only hits (n = 234), “CON”-tagged proteins (n = 124), and non-expressed proteins (n = 10). Centered log-ratio (CLR) was used to transform the data prior to applying ordinary least squares (OLS) regression for DA analysis. Comparisons were made between pre-and post-mission conditions in two ways: (i) The complete dataset (n = 160; 80 pre, 80 post) and (ii) within each of the 20 donors (n = 8 pre donor; 4 pre, 4 post). Proteins were classified as differentially abundant if they met the significance threshold using log_2_ fold change (log_2_FC), of |log_2_FC| > 1.5 and False Discovery Rate (FDR) < 0.05. Multiple testing correction was applied using the Benjamini-Hochberg procedure.

### 2.3 Modeling

Two model types from the python package scikit-learn (version 1.6.0) were used for modeling: logistic regression (specifically classification) and random forest. Default hyperparameter values were used. These distinct types were chosen as logistic regression boasts high interpretability and can learn linear relationships while random forest is an ensemble of decision trees that can learn nonlinear patterns in the data. Model performance was evaluated using area under the curve (AUC).

Models were trained to accomplish a binary classification task, where samples were classified as either pre-mission or post-mission samples. The intent was to have a simple model design that enabled the model to learn biological patterns indicative of readiness. Two objectives were defined to assess different readiness inference capabilities of the data. Objective 1 tested pattern recognition within the same donor by withholding 25% of replicates at random for testing. Here, “replicates” refers to biological replicates as repeated saliva samples were collected from the same donor across timepoints. No technical replicates (repeated measurements of the same sample) were used. Objective 2 tested model generalizability across donors by withholding all samples from 25% of donors for testing.

### 2.4 Feature importance

To assess the contribution of each feature to model performance, feature importance values were extracted or inferred from each model. Feature relevance was inferred from logistic classification models by extracting model coefficients, while gini scores were extracted from random forest models to indicate feature importance. Features were sorted by the absolute value of model coefficients or gini scores. From this sorted list, the features with the largest values were stored as top predictive features and were extracted for each model in the ten-fold cross-validation. To assess the statistical significance of extracted features, a binomial test was performed that evaluated whether a particular feature was deemed a predictive feature for a statistically significant number of models. Statistical significance was defined as *P* < 0.05.

## 3 Results

### 3.1 Traditional differential abundance fails to identify readiness markers from proteomics

One common CLS method for identifying biomarkers is the assessment of differentially abundant proteins that are common across individuals. Here, we asked whether this approach could predict readiness using the proteomics dataset collected by McKetney *et al.* where a less strict analysis of differential expression found 302 proteins significantly altered when comparing pre-mission to mission/post-mission samples (McKetney *et al.* 2022).

The initial proteomics dataset contained 2413 proteins. Altogether, we removed 349 entries (See Methods) resulting in a dataset containing 2064 protein entries for downstream analysis. Comparisons assessing differential abundance were made between pre- and post-mission conditions in two ways: (i) The complete dataset (n = 160 samples; 80 pre, 80 post) and (ii) within each of the 20 donors (n = 8 samples per donor; 4 pre, 4 post). The first method aligns with how differential abundance was assessed previously by McKetney *et al.* Across the complete dataset, the analysis identified two proteins as differentially abundant with thresholds of |log2FC| > 1.5 and FDR < 0.05. Within each donor, the analysis identified 78 total proteins differentially abundant with the same thresholds. Only 16 of the 20 donors had more than 1 differentially abundant protein. Of the 78 proteins, five proteins were differentially abundant within two different donors. These five proteins were not differentially abundant in the complete dataset. A binomial test revealed that observing the same protein in 2 of 20 donors was no more frequent than expected by chance (*P* > 0.05). These findings suggest that traditional DA methods lack sufficient power or generalizability to identify robust readiness biomarkers from this proteomics dataset.

### 3.2 Machine learning models can predict readiness using proteomics data

We next investigated whether ML models could achieve high predictive accuracy when distinguishing between pre- and post-mission. Additionally, we were interested in comparing these results to those achieved through LDA (McKetney *et al.* 2022). To best compare to and expand upon the published results, we designed two ML objectives to assess different readiness inference capabilities. Objective 1 tested the model’s readiness prediction on known donors by withholding 25% of replicates at random for testing. This objective and the LDA leave-one-out approach are most similar, as they both involve holding out replicates, so we use these results to compare ML and CLS predictive performance. We then designed a second objective that simulates a real-world deployment scenario in which the model must infer readiness for entirely new donors, ultimately evaluating model generalizability. Objective 2 assesses this by withholding all replicates from 25% of donors for testing. Both objectives were trained to classify pre- and post-mission samples (ready vs not-ready).

**Figure 2:**
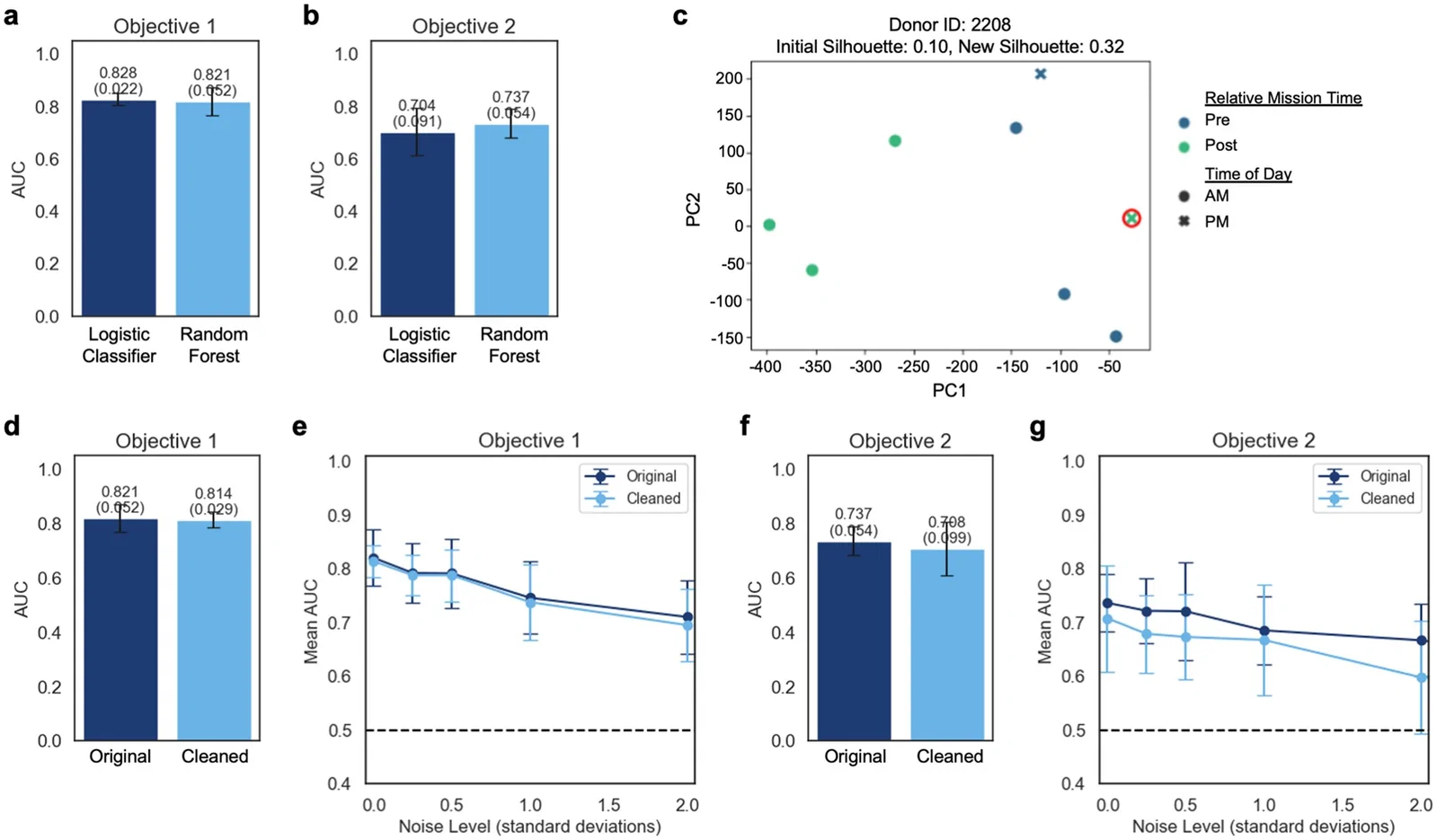
ML models predict readiness using proteomics data. (a) Objective 1 Mean AUC with standard deviation is plotted for both logistic classifier and random forest model types. (b) Objective 2 Mean AUC with standard deviation is plotted for both logistic classifier and random forest model types. (c) An example of a poorly correlated replicate that was removed using silhouette score calculations. (d) Comparison of Objective 1 random forest model performance using original dataset or cleaned dataset (where poorly correlated replicates have been removed). (e) Uncertainty quantification results for Objective 1 models are shown, plotting the mean AUC and standard deviation as error bars for various noise levels. (f) Comparison of Objective 2 random forest model performance using original dataset or cleaned dataset (where poorly correlated replicates have been removed). (g) Uncertainty quantification results for Objective 2 models are shown, plotting the mean AUC and standard deviation as error bars for various noise levels.

Initial proteomics modeling was performed using all samples and features available. Logistic classifier and random forest models were trained and evaluated using 10-fold cross validation to ensure robustness. The logistic classifier performed slightly better (AUC = 0.828 ± 0.022) than the random forest (AUC = 0.821 ± 0.052) for Objective 1. Notably, this performance is slightly better than that achieved by the 5-protein LDA model, which had an AUC = 0.771 (McKetney *et al.* 2022). For Objective 2, the random forest performed best (AUC = 0.737 ± 0.054) when compared to the logistic classifier (AUC = 0.704 ± 0.091) (Fig. 2a and b). Because the ML model was able to achieve an AUC > 0.7 for Objective 2, that indicates that the model was able to generalize to unseen donors. Together, these results highlight the promise of ML-based approaches in proteomics modeling.

To improve model performance, we next removed poorly correlated replicates from the dataset to allow the models to focus on true biological signals. We applied principal component analysis (PCA), reducing the data to two dimensions while capturing 66% of the variance, and then assessed replicate consistency by calculating the silhouette scores per donor using the pre- and post-mission groups. Replicates were marked for removal if their exclusion improved the per donor cluster silhouette score by 50% or more, a conservative threshold selected to prioritize only those removals likely to reflect substantial improvements in within-donor consistency, an example of which is shown in Fig. 2c. We identified and removed 29 poorly correlated replicates from the proteomics dataset. Using the “cleaned” dataset and all features, we retrained and tested the ML models to compare to the “original” dataset. Our results show similar model performance with decreased uncertainty, as indicated by a decrease in standard deviation (Objective 1 “original” AUC = 0.821 ± 0.052, “cleaned” AUC = 0.814 ± 0.029; Objective 2 “original” AUC = 0.737 ± 0.054, “cleaned” AUC = 0.708 ± 0.099; Fig. 2d and f). This highlights that the removal of poorly correlated replicates improves the stability of model performance.

We next performed an uncertainty quantification to examine model reliability and evaluate the tradeoff between predictive power and generalizability. Gaussian noise was incrementally applied to each protein and models were then evaluated using the “original” or “cleaned” feature sets. Model performance shifts were assessed using a t-test to compare the mean AUC across 10 cross-validation folds to the baseline, noise-free model (σ = 0). Our results show that Objective 1 models for both datasets exhibited a significant decrease in performance (original dataset *P* < 0.001; cleaned dataset *P* = 0.0242) when one standard deviation of noise is added per feature (σ = 1) (Fig. 2e). Objective 2 models for both datasets display a significant decrease in performance (original dataset *P* = 0.0389; cleaned dataset *P* = 0.00113) when two standard deviations of noise are added per feature (σ = 2) (Fig. 2g). This analysis revealed that the ML approach displayed strong resilience to moderate noise (σ = 0.5), but degraded under higher noise levels, reinforcing its robustness in realistic conditions. A direct comparison of CLS robustness to noise was not performed and its relative robustness should be assessed in future work.

### 3.3 ML approach identifies biomarkers, reveals minimal overlap with CLS-informed biomarkers, and enhances readiness predictions from proteomics data

**Figure 3:**
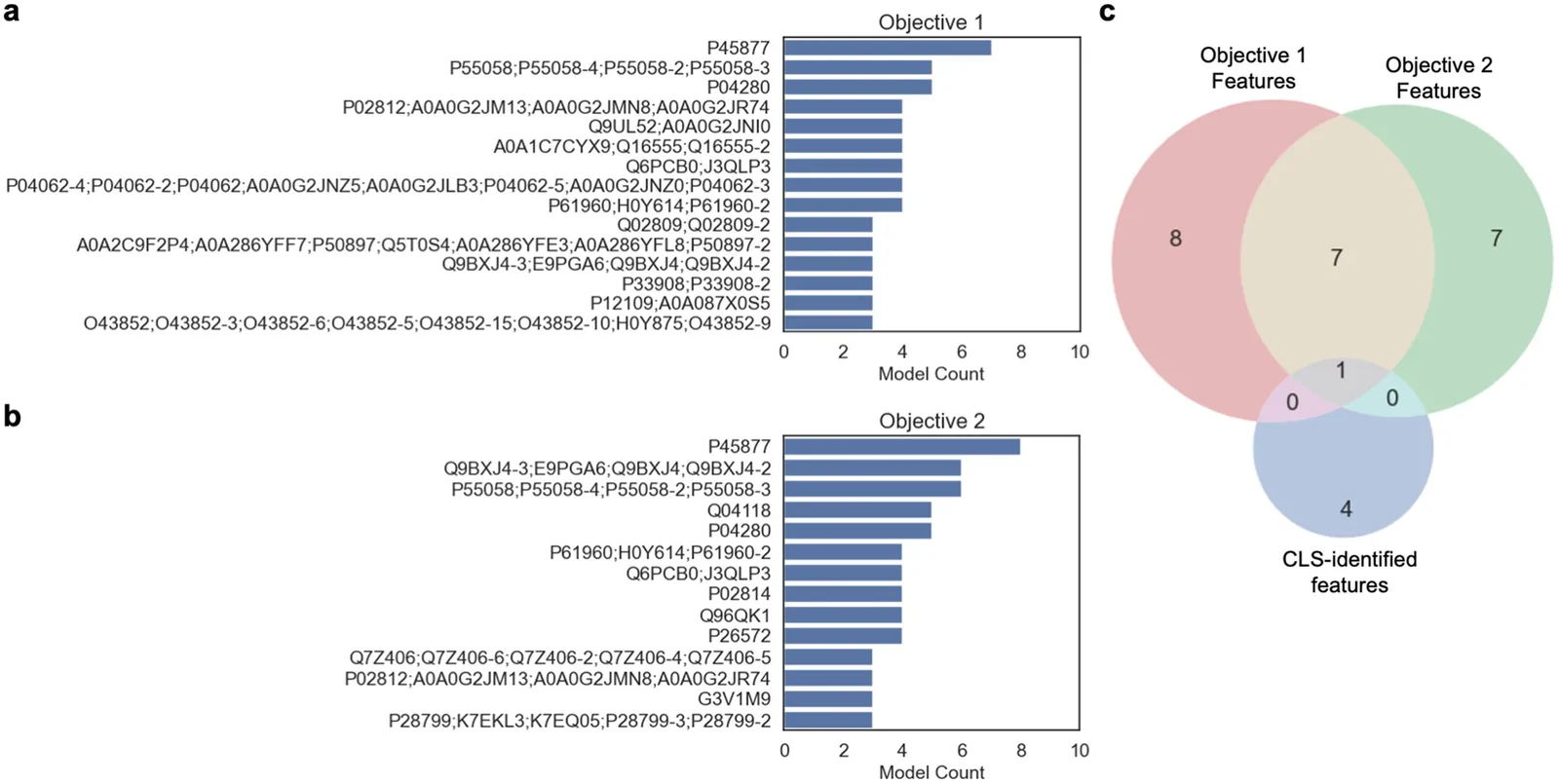
Feature extraction and comparison across techniques. (a, b) Extracted features that were in the top 20 important features across a statistically significant number of models are shown with the number of models that they were present in; Objective 1 (a) and Objective 2 (b). (c) Overlap of features identified through ML or CLS approaches.

The modeling efforts were designed to elucidate the proteins contributing to readiness so that biomarkers indicative of readiness could be identified. Further, we aimed to understand the overlap between these features and those identified in the McKetney *et al.* study (McKetney *et al.* 2022). We extracted the Gini Scores for each of the 10 cross-fold validated Random Forest models and used a binomial test to determine significance. A binomial test was applied to determine the minimum number of fold appearances required for statistical significance. We identified 15 significant features in the Objective 1 Random Forest Model and 14 significant features in the Objective 2 Random Forest model (Fig. 3a–c; Tables S1 and S2). This highlights that despite the challenges of donor-level generalization in Objective 2, the model consistently identified a stable set of key proteins, reinforcing the robustness of ML-based feature selection. Furthermore, eight features were found important for both Objective 1 and 2 (Fig. 3c), suggesting that there is a core subset of features essential for readiness prediction both within and across donors.

Since the identified features are proteins containing signals used to predict a state of readiness, we interpreted these features as biomarkers indicative of readiness. We compared the ML-identified features to the features identified through CLS approaches. Notably, of the five discriminators identified through LDA (McKetney *et al.* 2022), only one overlapped with the set of biomarkers identified through ML: the protein UFM1 (Fig. 3c). This overlap suggests that while CLS and ML approaches can identify common markers, ML methods capture alternative predictive features that were not previously prioritized or that are associated with complex, nonlinear patterns in the data that are not identified through CLS approaches.

**Figure 4:**
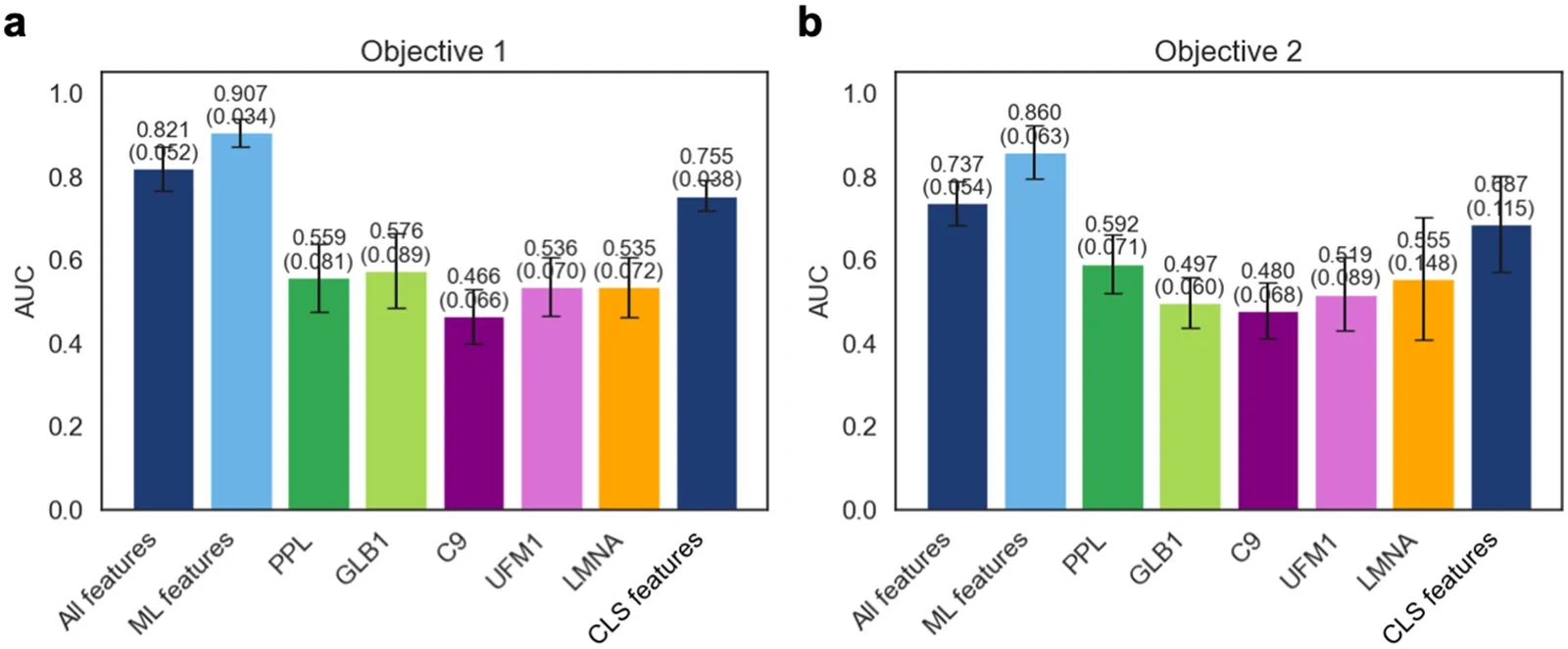
Modeling using ML-identified features enhances model performance. (a, b) Mean AUC with standard deviation is plotted for models trained/tested using various subsets of features for Objective 1 (a) and Objective 2 (b).

To better understand whether the identified biomarkers can predict readiness without other input signals, we trained new models using either ML-identified features or CLS-identified features. This strategy aimed to integrate prior biological knowledge into the ML framework while maintaining methodological rigor to improve interpretability and performance. While we recognize that this introduces data leakage, we use to serve as a validation for the markers identified (similar to the modeling in the McKetney *et al.* paper). Our results showed that models using ML-identified features achieved the highest performance, with Objective 1 reaching an AUC of 0.907 ± 0.034 and Objective 2 reaching an AUC of 0.860 ± 0.063 (Fig. 4a and b). These models outperformed those trained using any other subset of features, highlighting the power of using biologically guided feature selection to enhance ML predictive accuracy.

### 3.4 ML techniques predict readiness from metabolomics data

Metabolomics data provide key insights into an individual’s physiological state, with recent studies identifying salivary metabolites altered by physical and cognitive stress (Zauber *et al.* 2012, Ra *et al.* 2014, Chojnowska *et al.* 2021, McKetney *et al.* 2022, Morgan *et al.* 2022). Here, we investigated whether ML approaches could distinguish between pre-mission and post-mission states or readiness using salivary metabolomics data from 20 individuals.

**Figure 5:**
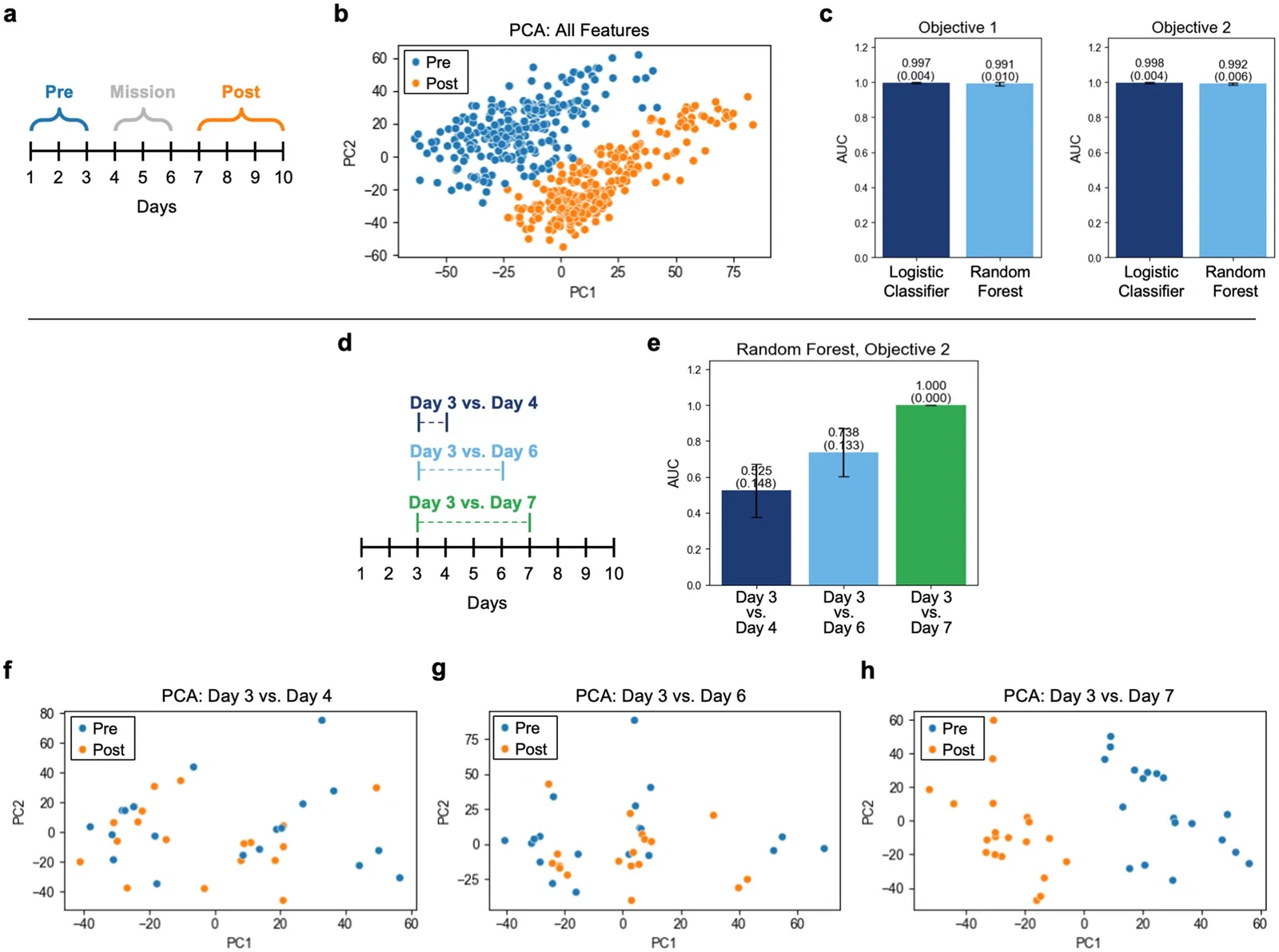
PCA and ML models can distinguish between states of readiness using metabolomics data. (a) Schematic showing the experimental timeline and separation of pre-mission, mission, and post-mission samples. (b) PC1/PC2 space reveals nearly linear separation between pre-mission and post-mission samples. (c) Mean AUC with standard deviation is shown for Objective 1 and Objective 2 models, where logistic classifier and random forest model types perform similarly. (d) Schematic showing the comparison of Day 3 sample with each of Days 4, 6, and 7. (e) Mean AUC with standard deviation is shown for Objective 2 random forest models for each temporal comparison. (f–h) PC1/PC2 spaces are shown for each temporal comparison.

The raw dataset collected by McKetney *et al.* (2022) contained 28 507 entries. To improve interpretability, we excluded entries with missing values (n = 6533) or without chemical name annotations (n = 22 151). Metabolites with identical chemical formulas were retained as unique features. Zero metabolites were identified as poorly correlated replicates through silhouette score calculations. The remaining dataset, containing 6356 metabolite entries, was normalized using log(x + 1) normalization and z-score standardization. For our first set of analyses, we grouped pre-mission samples from days 1–3 and post-mission samples from days 7–10 (Fig. 5a).

We first applied principal component analysis (PCA) to reduce dimensionality and identify dominant patterns. A near-linear separation between pre- and post-mission states in the PC1/PC2 space (Fig. 5b) suggested strong readiness-related signals in the data.

We then trained logistic classification and random forest models to predict readiness (Objectives 1 and 2). Using 10-fold cross-validation to reduce overfitting, both models achieved high AUC values using the full feature set (Fig. 5c). From this point forward, we focused on random forest models due to their utility for feature importance analysis.

To validate that our models were not overfitting or relying on signals that are not truly indicative of readiness, we developed a temporal validation strategy. New models were trained to classify between Day 3 (pre-mission) and Days 4, 6, and 7 individually (mission or post-mission) (Fig. 5d). The samples used were collected during the same time of day to control for metabolome profile changes that occur throughout a single day (Kawanishi *et al.* 2019). We hypothesized that model performance would be the lowest when comparing pre-mission samples, as no stressor had been encountered, and hypothesized that model performance would be highest when comparing to the post-mission timepoint on Day 7, as the mission had been completed. Indeed, we found that AUC values gradually increased for each comparison, with Day 3 vs. Day 4 achieving the lowest AUC (0.525 ± 0.148) and Day 3 vs Day 7 achieving the highest AUC (1.0 ± 0.0) (Fig. 5e). Further, we find that the PCA results for each of these comparisons correlates with model performance. This temporal pattern confirms that both unsupervised (PCA) and supervised (ML) methods can detect biologically meaningful signals tied to readiness.

### 3.5 ML approach matches model performance using metabolomics data

With a validated ML model capable of distinguishing between “ready” and “not ready” states, we next sought to identify potential metabolite biomarkers of readiness. Feature importance values were extracted from each of the 10 cross-validated models for both Objective 1 and Objective 2 using Gini scores and a binomial test.

**Figure 6:**
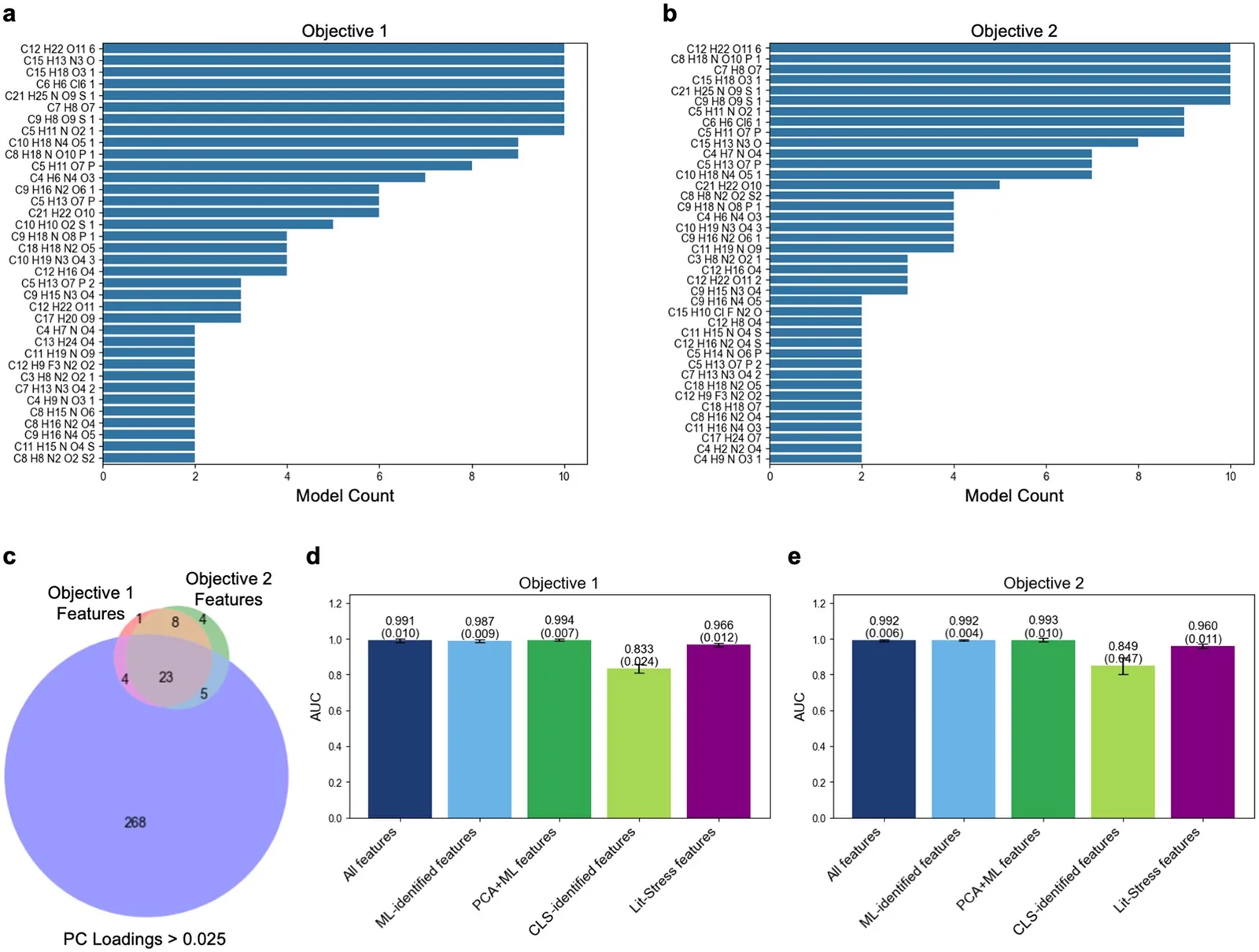
Models trained with subsets of ML-identified features perform similar to models trained using all features. (a, b) Extracted features that were in the top 20 important features across a statistically significant number of models are shown with the number of models that they were present in; Objective 1 (a) and Objective 2 (b). (c) Overlap of features identified through ML models or PC loadings. (d, e) Mean AUC with standard deviation is plotted for models trained/tested using various subsets of features for Objective 1 (d) and Objective 2 (e).

We assessed consistency across models by counting how often each metabolite appeared among the top 20 most important features across 10 model iterations. A binomial test was applied to these feature counts to identify statistically significant features, correcting for multiple comparisons using Benjamini-Hochberg FDR correction, with significance defined as an adjusted *P*-value < 0.05. This analysis yielded 36 statistically significant features for Objective 1 and 40 statistically significant features for Objective 2 (Fig. 6a and b; Tables S3 and S4), representing metabolite biomarker candidates consistently predictive of readiness across individuals. Of these model-identified features, eight overlapped between Objective 1 and Objective 2 (Fig. 6c).

Since the CLS methods (PCA and LDA) and modern ML models were capable of distinguishing between states of readiness, we next wanted to compare the biomarkers identified by each approach. The ML-identified biomarkers were the set of 23 features that overlap between Objective 1 and Objective 2. We extracted PC1 and PC2 loadings with values >0.025, extracting 20 PC1 loadings and 280 PC2 loadings which comprised our set of 300 PCA-identified biomarkers. Comparison of PCA-identified features to each set of ML model Objective features revealed that 23 features overlapped among all three groups (Fig. 6c). Of the five metabolite biomarkers identified through LDA (McKetney *et al.* 2022), zero overlapped with PCA or ML-identified features. These results demonstrate that the choice of analytical approach substantially impacts biomarker identification. Although some overlap in selected features was observed across methods, the broader differences suggest that each approach is capturing distinct underlying patterns in the data. For ML models, it is likely that the divergence in biomarker selection stems from the ability to detect nonlinear relationships that CLS methods may miss.

Finally, we investigated whether the use of a subset of features for ML model training would produce a high-performing model, as this smaller subset of features is more likely to be obtained from multi-plex sensors. We used four subsets of features: overlapping Objective 1 and Objective 2 features (ML-identified), overlapping ML-identified and PCA-identified features (PCA + ML features), CLS-identified features, and features associated with stress as identified by a literature review (Lit-Stress features). The Lit-Stress features included cortisol, glutamate, GABA, glycine, adrenaline, noradrenaline, serotonin, dopamine, and tryptophan. Our results showed that models trained using all features, ML-identified features, or PCA + ML features performed the best (Fig. 6d and e). Interestingly, the CLS-identified features resulted in models performing with AUCs of 0.833 ± 0.024 and 0.849 ± 0.047 (Objective 1 and 2; Fig. 6d and e), which is higher than the performance of the LDA model using the same subset of features (McKetney *et al.* 2022). The Lit-Stress feature model also achieved high performance (Objective 1 AUC = 0.966 ± 0.012, Objective 2 AUC = 0.960 ± 0.011), validating the biological-realness of the modeling approach and predictive capabilities. Altogether, these results validate the metabolomics ML models and present new biomarkers indicative of readiness that have not been previously associated with stress.

## 4 Discussion

The use of ML approaches enables enhancement and validation of predictions of readiness. Here, we show that ML analysis pipelines applied to proteomics or metabolomics data produce models capable of predicting readiness. Proteomics ML models using all features achieved AUCs of 0.821 ± 0.052 and 0.737 ± 0.055 for Objectives 1 and 2. After extracting features to select biomarkers indicative of readiness, we identified only one protein biomarker that overlapped with the biomarkers identified in the previously published study (McKetney *et al.* 2022). Notably, the use of only ML-identified biomarkers to train and test new proteomics models improved predictive performance for both objectives (Objective 1 AUC = 0.907 ± 0.034 and Objective 2 AUC = 0.860 ± 0.063). We applied a similar ML approach to the metabolomics dataset as well as the CLS technique, PCA. The near-linear boundary in the PC1/PC2 space separating states of readiness indicated that this approach was able to distinguish between states of readiness with high accuracy. To continue to understand and compare CLS vs. modern ML performance and biomarker selection, we trained ML models on metabolomics data. These models were able to predict readiness, where both logistic classification and random forest models for Objectives 1 and 2 produced AUC > 0.99. We found some overlap of ML-identified and PCA-identified biomarkers, but no overlap with biomarkers previously identified using LDA (McKetney *et al.* 2022). Finally, random forest models trained using subsets of features demonstrated high (AUC > 0.99) performance when using random forest model- and PCA-identified features, high performance using Lit-Stress features (AUC = 0.966, AUC = 0.960), and good performance using CLS-identified features (Objective 1 AUC = 0.833 ± 0.024, Objective 2 AUC = 0.849 ± 0.047). Altogether, these results demonstrate the power of a ML analysis pipelines for selecting robust biomarkers and training ML models capable of predicting states of readiness with high accuracy.

### 4.1 Limitation of binary readiness labels

The dataset used in this paper is from the most comprehensive assessment of readiness, implicating that highly relevant biological insights can be garnered from the results. While this is true, we would like to point out that readiness had to be assumed and defined in a binary manner–where samples were assigned with “ready” or “not ready” labels based on whether they were collected before or after mission completion. Because data collection did not involve assessments that would enable calculation of a true readiness score, this assumption is an innate and unavoidable flaw. We recognize the flaws of this assumption, acknowledge that it limits the interpretation of the results, and propose the collection of a similar dataset with a continuous scale of readiness scores to validate the findings presented in this paper.

### 4.2 ML approach enhances predictive power of single -omics models

It is important to note that the feature-subset models introduce data leakage, as feature selection used the full dataset prior to splitting into train and test subsets. This concern is discussed in this section. The comparison of ML modeling and CLS methods is difficult due to key differences in data handling and validation. In the study that published the dataset used in this paper, LDA was applied using leave-one-out across the dataset to identify top proteins and metabolites indicative of readiness. While this CLS approach uses all data available, our ML models for Objectives 1 and 2 strictly hold out subsets of data for validation. This approach for the ML modeling ensures a rigorous evaluation of generalizability to new samples and donors. Further, our approach incorporates 10-fold cross-validation to mitigate overfitting and reinforcing reliability to perform on unseen data.

The ML pipeline using a subset of features for training introduces data leakage as the entire dataset contributed to feature selection. Because there were only 20 individuals included in the dataset, we did not want to reduce the number of samples any further to have an independent dataset for testing. However, leakage was minimized by performing 10-fold cross-validation before feature extraction and using a binomial test to select features with high stability across folds. We think this small amount of leakage is justified because here; we are using these models to generate hypotheses about which features (proteins or metabolites) may contain signals that are indicative of readiness. As there is leakage in this model, it will be necessary to collect and use an independent dataset to validate the identified features.

The LDA leave-one-out approach is most comparable to our Objective 1, as both approaches held out replicates instead of donors, so we focus on Objective 1 ML model results here. Using the proteomics dataset, the model produced from the ML approach using a subset of features achieved the highest predictive performance (AUC = 0.907), followed by the initial ML models (AUC = 0.83 and AUC = 0.821), and then the LDA approach (AUC = 0.771). For the metabolomics dataset, the initial ML models and the model produced from the ML approach using a subset of features performed best (AUC > 0.99) when compared to the LDA approach (AUC = 0.871). Despite the absence of feature engineering or selection and the stricter validation criteria, the Objective 1 ML model performed on par with the McKetney *et al.* CLS approach. These results highlight the power of using a ML approach to predict states readiness.

While the Objective 2 model results cannot be directly compared to those of the CLS results, this modeling approach simulates a real-world deployment scenario in which the model must infer readiness for entirely new donors. As such, performance in Objective 2 serves as a stronger indicator of the model’s ability to adapt to novel cases and underscores the increased difficulty and utility compared to Objective 1 and CLS Analysis. Notably, the Objective 2 ML model, was able to generalize to some unseen donors, exhibiting strong performance. These results demonstrate the improved predictive power and generalizability of single-omic ML models that are supplemented with biologically informed feature selection, as opposed to approaches that use solely CLS or ML methods.

In addition to evaluating model performance, we also assessed model robustness by performing an uncertainty quantification on the proteomics models and a temporal assessment for the metabolomics models. The uncertainty quantification revealed that proteomics models are insensitive to biologically realistic levels of noise (σ < 1, after normalization) and sensitive to higher levels of noise, demonstrating model robustness. The temporal assessment of metabolomics models suggested that these models are learning patterns indicative of readiness, as model performance increased as readiness decreased.

As the metabolomics model achieved near-perfect AUC while the proteomics model achieved a highest AUC of 0.907, it begs the question of whether both modes of data are necessary for readiness predictions. The near-perfect metabolomics model AUC suggests that metabolomics alone may be sufficient for readiness classification, however further validation with an independent is necessary to confirm that this near-perfect performance is not due to overfitting. Further, metabolomics performance may reflect confounding variables such as strong mission-related metabolic shifts, time-of-day effects, or dietary changes. While the first two possible confounds are partially addressed by temporal validation analyses, new data collection in a cohort where diet is controlled is required to address concerns about diet influencing the metabolomics model. Finally, the use of only a metabolomics model likely limits the biological understanding of readiness. Proteomics provides complementary mechanistic interpretation at the protein level, which may be critical for understanding the biology of cognitive and physical readiness. Further, as the metabolomics model exhibits a risk of overfitting, proteomics-based models may serve as a more conservative and interpretable foundation for readiness assessment until independent validation is performed.

### 4.3 Explainable ML models align with biology and reveal key biomarkers indicative of readiness

The use of explainable ML model types offers interpretable pathways to identify features that most contribute to model predictions, enhancing biological insights (Elsborg and Salvatore 2023, Kumar and Das 2023, Wang and Ji 2025). Here, we used an ML approach to extract subsets of protein and metabolite features that strongly contribute to predictions of readiness. Both the metabolomics and proteomics models appear to be driven by a small number of features (Figs. 3a and b and 6a and b). Notably, we found that training new models using only these significant features resulted in equal or increased performance when compared to models trained on all features. This suggests that the selected features are not only computationally informative, but also biologically meaningful.

To further assess the biological relevance of identified features, we performed a literature review of biological function and associated conditions. We found that 10 out of 33 proteins identified through ML in this study have been previously associated with physical or cognitive stress mechanisms, being tied to functions such as oxidative stress responses, antioxidant properties, endoplasmic reticulum stress, muscle function, and cognitive function (Table S5). In contrast, allantoin was the only identified metabolites for which a link to physical stress has been published (Tsahar *et al.* 2006, Hasikova *et al.* 2020). The metabolomics results also underscore the importance of controlling as many variables as possible during studies as several of the extracted metabolites are not thought to be endogenous to human saliva and likely came from consumption of food or medications (i.e. Methoxsalen, 1-Deoxy-D-xylulose 5-phosphate, 2-C-Methyl-D-erythritol 4-phosphate). The remaining human-relevant biomarkers present a novel opportunity to enhance our understanding of the biological basis of readiness. Altogether, this work presents several novel biomarkers of readiness that require follow-up wet-lab experiments to determine how they fit into the biological picture of readiness, which will validate them as biomarkers.

## Supplementary Material

btag585_Supplementary_Data

## Code availability

The analysis pipelines are provided as Jupyter notebooks, including all code and documentation, and are available publicly on GitHub at {https://github.com/netrias/ReadinessClassification}.
