# Supplementary material for: Comparison of classic statistical methods and machine learning approaches to classify readiness
Source: Bioinformatics. 2026 Aug 25;42(9):btag585. doi: 10.1093/bioinformatics/btag585 (PMC13533562; doi:10.1093/bioinformatics/btag585)
Supplement: Supplementary file 1 [file btag585_supplementary_data.docx]

**Supplementary Information**

| **Feature** | **Protein Name** | **Model Count** | **Adjusted p-value** |
| --- | --- | --- | --- |
| P45877 | Peptidyl-prolyl cis-trans isomerase C | 7 | 1.08e-10 |
| P55058;P55058-4;P55058-2;P55058-3 | Phospholipid transfer protein | 5 | 2.38e-06 |
| P04280 | Basic salivary proline-rich protein 1 | 5 | 2.38e-06 |
| P02812;A0A0G2JM13;A0A0G2JMN8;A0A0G2JR74 | Basic salivary proline-rich protein 2 | 4 | 2.04e-04 |
| Q9UL52;A0A0G2JNI0 | Transmembrane protease serine 11E | 4 | 2.04e-04 |
| A0A1C7CYX9;Q16555;Q16555-2 | Dihydropyrimidinase-related protein 2 | 4 | 2. 04e-04 |
| Q6PCB0;J3QLP3 | von Willebrand factor A domain-containing protein | 4 | 2. 04e-04 |
| P04062-4;P04062-2;P04062;  A0A0G2JNZ5;A0A0G2JLB3;P04062-5;  A0A0G2JNZ0;P04062-3 | Glucosylceramidase | 4 | 2.04e-04 |
| P61960;H0Y614;P61960-2 | Ubiquitin-fold modifier 1 | 4 | 2.04e-04 |
| Q02809;Q02809-2 | Procollagen-lysine,2-oxoglutarate 5-dioxygenase 1 | 3 | 1.38e-02 |
| A0A2C9F2P4;A0A286YFF7;P50897;Q5T0S4;A0A286YFE3;A0A286YFL8;P50897-2 | Palmitoyl-protein thioesterase 1 | 3 | 1.38e-02 |
| Q9BXJ4-3;E9PGA6;Q9BXJ4;Q9BXJ4-2 | Complement C1q tumor necrosis factor-related protein | 3 | 1.38e-02 |
| P33908;P33908-2 | Mannosyl-oligosaccharide 1,2-alpha-mannosidase IA | 3 | 1.38e-02 |
| P12109;A0A087X0S5 | Collagen alpha-1(VI) chain | 3 | 1.38e-02 |
| O43852;O43852-3;O43852-6;O43852-5;O43852-15;O43852-10;H0Y875;O43852-9 | Calumenin | 3 | 1.38e-02 |

**Supplementary Table S1. Extracted features from proteomics Objective 1 model.** Table includes feature entry, associated protein name, number of models (out of 10) where this protein was included in the list of top 20 important proteins, and adjusted p-value from binomial test.

| **Feature** | **Protein Name** | **Model Count** | **Adjusted p-value** |
| --- | --- | --- | --- |
| P45877 | Peptidyl-prolyl cis-trans isomerase C | 8 | 4.43E-13 |
| Q9BXJ4-3;E9PGA6;Q9BXJ4;Q9BXJ4-2 | Complement C1q tumor necrosis factor-related protein | 6 | 1.94E-08 |
| P55058;P55058-4;P55058-2;P55058-3 | Phospholipid transfer protein | 6 | 1.94E-08 |
| Q04118 | Basic salivary proline-rich protein 3 | 5 | 2.38E-06 |
| P04280 | Basic salivary proline-rich protein 1 | 5 | 2.38E-06 |
| P61960;H0Y614;P61960-2 | Ubiquitin-fold modifier 1 | 4 | 2.04E-04 |
| Q6PCB0;J3QLP3 | von Willebrand factor A domain-containing protein | 4 | 2.04E-04 |
| P02814 | Submaxillary gland androgen-regulated protein | 4 | 2.04E-04 |
| Q96QK1 | Vacuolar protein sorting-associated protein 35 | 4 | 2.04E-04 |
| P26572 | Alpha-1,3-mannosyl-glycoprotein 2-beta-N-acety… | 4 | 2.04E-04 |
| Q7Z406;Q7Z406-6;Q7Z406-2;Q7Z406-4;Q7Z406-5 | Myosin-14 | 3 | 1.38E-02 |
| P02812;A0A0G2JM13;A0A0G2JMN8;A0A0G2JR74 | Basic salivary proline-rich protein | 3 | 1.38E-02 |
| G3V1M9 | NaN | 3 | 1.38E-02 |
| P28799;K7EKL3;K7EQ05;P28799-3;P28799-2 | Granulins;Acrogranin;Paragranulin;Granulin-1 | 3 | 1.38E-02 |

**Supplementary Table S2. Extracted features from proteomics Objective 2 model.** Table includes feature entry, associated protein name, number of models (out of 10) where this protein was included in the list of top 20 important proteins, and adjusted p-value from binomial test.

| **Feature** | **Metabolite Name** | **Model Count** | **Adjusted p-value** |
| --- | --- | --- | --- |
| C12 H22 O11 6 | Sucrose | 10 | 4.57E-24 |
| C7 H8 O7 | 2,6-Anhydro-6-carboxy-3-deoxyhex-2-enonic acid | 10 | 4.57E-24 |
| C15 H18 O3 1 | Loxoprofen | 10 | 4.57E-24 |
| C6 H6 Cl6 1 | lindane | 10 | 4.57E-24 |
| C21 H25 N O9 S 1 | 1-(3,4-Dimethoxyphenyl)-2-{[(2E)-3-(3,4-dimethoxyphenyl)-2-propenoyl]amino}ethyl hydrogen sulfate | 10 | 4.57E-24 |
| C9 H8 O9 S 1 | 3-[3,5-Dihydroxy-4-(sulfooxy)phenyl]-2-oxopropanoic acid | 10 | 4.57E-24 |
| C5 H11 N O2 1 | 5-Aminopentanoate | 10 | 4.57E-24 |
| C15 H13 N3 O | MFCD00800998 | 10 | 4.57E-24 |
| C10 H18 N4 O5 1 | Gln-Gln | 9 | 1.45E-20 |
| C8 H18 N O10 P 1 | N-Gluconyl ethanolamine phosphate | 9 | 1.45E-20 |
| C5 H11 O7 P | 1-Deoxy-D-xylulose 5-phosphate | 8 | 2.06E-17 |
| C4 H6 N4 O3 | (S)-(+)-Allantoin | 7 | 1.74E-14 |
| C5 H13 O7 P | 2-C-Methyl-D-erythritol 4-phosphate | 6 | 9.68E-12 |
| C21 H22 O10 | 3-O-{4-[3-(1-Carboxyethyl)phenoxy3-O-{4-[3-(1-Carboxyethyl)phenoxy]phenyl}-D-glucopyranuronic acid]phenyl}-D-gl... | 6 | 9.68E-12 |
| C9 H16 N2 O6 1 | glu-thr | 6 | 9.68E-12 |
| C10 H10 O2 S 1 | 3238 | 5 | 3.68E-09 |
| C10 H19 N3 O4 3 | Leucylasparagine | 4 | 9.73E-07 |
| C9 H18 N O8 P 1 | (R)-4'-Phosphopantothenate | 4 | 9.73E-07 |
| C12 H16 O4 | ETHYL VANILLIN PROPYLENE GLYCOL ACETAL | 4 | 9.73E-07 |
| C18 H18 N2 O5 | 3-[2-(Hydroxymethyl)-4-methoxyphenyl]-6-methoxy-4-oxo-3,4-dihydro-1(2H)-quinazolinecarbaldehyde | 4 | 9.73E-07 |
| C17 H20 O9 | 1,3,4-Trihydroxy-5-{[(2Z)-3-(3-hydroxy-4-methoxyphenyl)-2-propenoyl]oxy}cyclohexanecarboxylic acid | 3 | 1.77E-04 |
| C9 H15 N3 O4 | asn-pro | 3 | 1.77E-04 |
| C5 H13 O7 P 2 | 2-C-Methyl-D-erythritol 4-phosphate | 3 | 1.77E-04 |
| C12 H22 O11 | Sucrose | 3 | 1.77E-04 |
| C11 H15 N O4 S | 1988595 | 2 | 2.10E-02 |
| C9 H16 N4 O5 | gln-asn | 2 | 2.10E-02 |
| C3 H8 N2 O2 1 | 3-Amino-L-alanine | 2 | 2.10E-02 |
| C8 H16 N2 O4 | Val-Ser | 2 | 2.10E-02 |
| C8 H15 N O6 | N-Acetyl-β-D-glucosamine | 2 | 2.10E-02 |
| C4 H9 N O3 1 | L-Threonine | 2 | 2.10E-02 |
| C7 H13 N3 O4 2 | Glycylglutamine | 2 | 2.10E-02 |
| C12 H9 F3 N2 O2 | Leflunomide | 2 | 2.10E-02 |
| C11 H19 N O9 | N-Acetyl-β-neuraminate | 2 | 2.10E-02 |
| C13 H24 O4 | Brassylic acid | 2 | 2.10E-02 |
| C4 H7 N O4 | L-Aspartate | 2 | 2.10E-02 |
| C8 H8 N2 O2 S2 | Thiolutin | 2 | 2.10E-02 |

**Supplementary Table S3. Extracted features from metabolomics Objective 1 model.** Table includes feature entry, associated metabolite name, number of models (out of 10) where this metabolite was included in the list of top 20 important features, and adjusted p-value from binomial test.

| **Feature** | **Metabolite Name** | **Model Count** | **Adjusted p-value** |
| --- | --- | --- | --- |
| C12 H22 O11 6 | Sucrose | 10 | 4.85E-24 |
| C7 H8 O7 | 2,6-Anhydro-6-carboxy-3-deoxyhex-2-enonic acid | 10 | 4.85E-24 |
| C15 H18 O3 1 | Loxoprofen | 10 | 4.85E-24 |
| C21 H25 N O9 S 1 | 1-(3,4-Dimethoxyphenyl)-2-{[(2E)-3-(3,4-dimethoxyphenyl)-2-propenoyl]amino}ethyl hydrogen sulfate | 10 | 4.85E-24 |
| C9 H8 O9 S 1 | 3-[3,5-Dihydroxy-4-(sulfooxy)phenyl]-2-oxopropanoic acid | 10 | 4.85E-24 |
| C8 H18 N O10 P 1 | N-Gluconyl ethanolamine phosphate | 10 | 4.85E-24 |
| C6 H6 Cl6 1 | lindane | 9 | 1.54E-20 |
| C5 H11 O7 P | 1-Deoxy-D-xylulose 5-phosphate | 9 | 1.54E-20 |
| C5 H11 N O2 1 | 5-Aminopentanoate | 9 | 1.54E-20 |
| C15 H13 N3 O | MFCD00800998 | 8 | 2.19E-17 |
| C10 H18 N4 O5 1 | Gln-Gln | 7 | 1.85E-14 |
| C4 H7 N O4 | L-Aspartate | 7 | 1.85E-14 |
| C5 H13 O7 P | 2-C-Methyl-D-erythritol 4-phosphate | 7 | 1.85E-14 |
| C21 H22 O10 | 3-O-{4-[3-(1-Carboxyethyl)phenoxy]phenyl}-D-glucopyranuronic acid | 5 | 3.91E-09 |
| C8 H8 N2 O2 S2 | Thiolutin | 4 | 1.03E-06 |
| C9 H18 N O8 P 1 | (R)-4'-Phosphopantothenate | 4 | 1.03E-06 |
| C9 H16 N2 O6 1 | glu-thr | 4 | 1.03E-06 |
| C11 H19 N O9 | N-Acetyl-β-neuraminate | 4 | 1.03E-06 |
| C4 H6 N4 O3 | (S)-(+)-Allantoin | 4 | 1.03E-06 |
| C10 H19 N3 O4 3 | Leucylasparagine | 4 | 1.03E-06 |
| C9 H15 N3 O4 | asn-pro | 3 | 1.88E-04 |
| C3 H8 N2 O2 1 | 3-Amino-L-alanine | 3 | 1.88E-04 |
| C12 H16 O4 | ETHYL VANILLIN PROPYLENE GLYCOL ACETAL | 3 | 1.88E-04 |
| C12 H22 O11 2 | Sucrose | 3 | 1.88E-04 |
| C4 H2 N2 O4 | alloxan | 2 | 2.23E-02 |
| C17 H24 O7 | 3,8,15-Trihydroxy-12,13-epoxytrichothec-9-en-4-yl acetate | 2 | 2.23E-02 |
| C11 H16 N4 O3 | His-pro | 2 | 2.23E-02 |
| C8 H16 N2 O4 | Val-Ser | 2 | 2.23E-02 |
| C12 H16 N2 O4 S | Cys-tyr | 2 | 2.23E-02 |
| C18 H18 O7 | 5,7-Dihydroxy-2-(3,4,5-trimethoxyphenyl)-4-chromanone | 2 | 2.23E-02 |
| C12 H9 F3 N2 O2 | Leflunomide | 2 | 2.23E-02 |
| C18 H18 N2 O5 | 3-[2-(Hydroxymethyl)-4-methoxyphenyl]-6-methoxy-4-oxo-3,4-dihydro-1(2H)-quinazolinecarbaldehyde | 2 | 2.23E-02 |
| C7 H13 N3 O4 2 | Glycylglutamine | 2 | 2.23E-02 |
| C5 H13 O7 P 2 | 2-C-Methyl-D-erythritol 4-phosphate | 2 | 2.23E-02 |
| C5 H14 N O6 P | (S)-?-glycerophosphorylethanolamine | 2 | 2.23E-02 |
| C9 H16 N4 O5 | gln-asn | 2 | 2.23E-02 |
| C15 H10 Cl F N2 O | N-Desalkylflurazepam | 2 | 2.23E-02 |
| C12 H8 O4 | Methoxsalen | 2 | 2.23E-02 |
| C11 H15 N O4 S | 1988595 | 2 | 2.23E-02 |
| C4 H9 N O3 1 | L-Threonine | 2 | 2.23E-02 |

**Supplementary Table S4. Extracted features from metabolomics Objective 1 model.** Table includes feature entry, associated metabolite name, number of models (out of 10) where this metabolite was included in the list of top 20 important features, and adjusted p-value from binomial test.

| **Protein Name** | **Associated with:** | **Citation** |
| --- | --- | --- |
| Complement C1q Tumor Necrosis Factor-Related Protein | Metabolic and oxidative stress; mood disorders | (Q. Chen et al., 2024; Petersen et al., 2016; Shi et al., 2024; Zhang et al., 2023) |
| Phospholipid Transfer Protein | Oxidative stress | (Desrumaux et al., 2010; Zhou et al., 2014) |
| Basic Salivary Proline-Rich Proteins 1-3 | Antioxidant properties | (Komatsu et al., 2020) |
| Ubiquitin-Fold Modifier 1 | Cellular stress | (McKetney et al., 2022) |
| Von Willebrand Factor A Domain-Containing Protein | Inflammation and oxidative stress | (J. Chen & Chung, 2018; Dal Lin et al., 2021) |
| Dihydropyrimidinase-Related Protein 2 | Mood disorders (anxiety, bipolar disorder) | (Guliyeva & Mekhtiev, 2023; Saia-Cereda et al., 2017) |
| Glucosylceramidase | Cellular stress | (Uchida et al., 2002) |
| Palmitoyl-Protein Thioesterase 1 | Cognitive stress | (Wu et al., 2019) |
| Collagen Alpha-1(VI) Chain | Physical readiness; muscular disorders | (Williams et al., 2022) |
| Calumenin | Cellular stress | (Lee et al., 2013) |

**Supplementary Table S5. Literature review of proteins associated with various forms of cognitive or physical stress.** Table includes protein name, a brief description of the relevant cognitive or physical stress function, and one or more citations.

**References**

Chen, J., & Chung, D. W. (2018). Inflammation, von Willebrand factor, and ADAMTS13. *Blood*, *132*(2), 141–147. https://doi.org/10.1182/blood-2018-02-769000

Chen, Q., Sun, T., He, Q., Yu, J., Zhang, X., Han, L., & Ren, Y. (2024). Study of decreased serum levels of C1q/TNF-related protein 4 (CTRP4) in major depressive disorder. *Journal of Psychiatric Research*, *172*, 274–280. https://doi.org/10.1016/j.jpsychires.2024.01.046

Dal Lin, C., Acquasaliente, L., Iliceto, S., De Filippis, V., Vitiello, G., & Tona, F. (2021). Von Willebrand Factor Multimers and the Relaxation Response: A One-Year Study. *Entropy*, *23*(4), Article 4. https://doi.org/10.3390/e23040447

Desrumaux, C., Deckert, V., Lemaire-Ewing, S., Mossiat, C., Athias, A., Vandroux, D., Dumont, L., Monier, S., Pais de Barros, J.-P., Klein, A., De Maistre, E., Blache, D., Beley, A., Marie, C., Garnier, P., & Lagrost, L. (2010). Plasma Phospholipid Transfer Protein Deficiency in Mice Is Associated With a Reduced Thrombotic Response to Acute Intravascular Oxidative Stress. *Arteriosclerosis, Thrombosis, and Vascular Biology*, *30*(12), 2452–2457. https://doi.org/10.1161/ATVBAHA.110.207654

Guliyeva, Sh. M., & Mekhtiev, A. A. (2023). Involvement of Dihydropyrimidinase-Related Protein 2 in Human Anxiety Regulation. *Journal of Evolutionary Biochemistry and Physiology*, *59*(1), 133–140. https://doi.org/10.1134/S0022093023010118

Komatsu, T., Kobayashi, K., Morimoto, Y., Helmerhorst, E., Oppenheim, F., & Chang-il Lee, M. (2020). Direct evaluation of the antioxidant properties of salivary proline-rich proteins. *Journal of Clinical Biochemistry and Nutrition*, *67*(2), 131–136. https://doi.org/10.3164/jcbn.19-75

Lee, J. H., Kwon, E. J., & Kim, D. H. (2013). Calumenin has a role in the alleviation of ER stress in neonatal rat cardiomyocytes. *Biochemical and Biophysical Research Communications*, *439*(3), 327–332. https://doi.org/10.1016/j.bbrc.2013.08.087

McKetney, J., C. Jenkins, C., Minogue, C., M. Mach, P., K. Hussey, E., G. Glaros, T., Coon, J., & S. Dhummakupt, E. (2022). Proteomic and metabolomic profiling of acute and chronic stress events associated with military exercises. *Molecular Omics*, *18*(4), 279–295. https://doi.org/10.1039/D1MO00271F

Petersen, P. S., Wolf, R. M., Lei, X., Peterson, J. M., & Wong, G. W. (2016). Immunomodulatory roles of CTRP3 in endotoxemia and metabolic stress. *Physiological Reports*, *4*(5), e12735. https://doi.org/10.14814/phy2.12735

Saia-Cereda, V. M., Cassoli, J. S., Martins-de-Souza, D., & Nascimento, J. M. (2017). Psychiatric disorders biochemical pathways unraveled by human brain proteomics. *European Archives of Psychiatry and Clinical Neuroscience*, *267*(1), 3–17. https://doi.org/10.1007/s00406-016-0709-2

Shi, L., Tan, Y., Zheng, W., Cao, G., Zhou, H., Li, P., Cui, J., Song, Y., Feng, L., Li, H., Shan, W., Zhang, B., & Yi, W. (2024). CTRP3 alleviates mitochondrial dysfunction and oxidative stress injury in pathological cardiac hypertrophy by activating UPRmt via the SIRT1/ATF5 axis. *Cell Death Discovery*, *10*(1), 1–14. https://doi.org/10.1038/s41420-024-01813-x

Uchida, Y., Murata, S., Schmuth, M., Behne, M. J., Lee, J. D., Ichikawa, S., Elias, P. M., Hirabayashi, Y., & Holleran, W. M. (2002). Glucosylceramide synthesis and synthase expression protect against ceramide-induced stress. *Journal of Lipid Research*, *43*(8), 1293–1302. https://doi.org/10.1194/jlr.M100442-JLR200

Williams, L., Layton, T., Yang, N., Feldmann, M., & Nanchahal, J. (2022). Collagen VI as a driver and disease biomarker in human fibrosis. *The FEBS Journal*, *289*(13), 3603–3629. https://doi.org/10.1111/febs.16039

Wu, Y., Zhang, Q., Qi, Y., Gao, J., Li, W., Lv, L., Chen, G., Zhang, Z., Yue, X., & Peng, S. (2019). Enzymatic activity of palmitoyl‐protein thioesterase‐1 in serum from schizophrenia significantly associates with schizophrenia diagnosis scales. *Journal of Cellular and Molecular Medicine*, *23*(9), 6512–6518. https://doi.org/10.1111/jcmm.14496

Zhang, H., Zhang-Sun, Z., Xue, C., Li, X., Ren, J., Jiang, Y., Liu, T., Yao, H., Zhang, J., Gou, T., Tian, Y., Lei, W., & Yang, Y. (2023). CTRP family in diseases associated with inflammation and metabolism: Molecular mechanisms and clinical implication. *Acta Pharmacologica Sinica*, *44*(4), 710–725. https://doi.org/10.1038/s41401-022-00991-7

Zhou, T., He, Q., Tong, Y., Zhan, R., Xu, F., Fan, D., Guo, X., Han, H., Qin, S., & Chui, D. (2014). Phospholipid transfer protein (PLTP) deficiency impaired blood–brain barrier integrity by increasing cerebrovascular oxidative stress. *Biochemical and Biophysical Research Communications*, *445*(2), 352–356. https://doi.org/10.1016/j.bbrc.2014.01.194
